# Enhanced Electrochromic Properties of NiO*_x_* Films Through Magnesium Doping Strategy

**DOI:** 10.3390/nano15161217

**Published:** 2025-08-08

**Authors:** Xiaoyu Yao, Shuai Ding, Xiaoyu Shen, Congkai Guo, Yao Liu, Wenjuan Xia, Guohua Wu, Yaohong Zhang

**Affiliations:** 1 School of Physics, Northwest University, Xi’an 710127, China; xiaoyuyao@stumail.nwu.edu.cn (X.Y.); m18145091036@163.com (S.D.); shenxiaoyu0119@163.com (X.S.); m13105430335@163.com (C.G.); yaoliu@stumail.nwu.edu.cn (Y.L.); xiawenjuan@stumail.nwu.edu.cn (W.X.); 2 Qingdao Innovation and Development Center of Harbin Engineering University, Harbin Engineering University, Qingdao 266000, China; 3 College of Materials Science and Chemical Engineering, Harbin Engineering University, Harbin 150001, China

**Keywords:** electrochromism, NiO*_x_* films, sol–gel method, Mg doping, oxygen defects

## Abstract

In order to improve the electrochromic properties of NiO*_x_* films, Mg ions were introduced into NiO*_x_* films using the sol–gel method and the spin-coating method. The introduction of Mg ions leads to the loose structure of the compact NiO*_x_* film, which can provide more channels for the transport of OH^−^. In addition, the introduction of Mg ions increases the oxygen vacancies and oxygen interstitial defects in the NiO*_x_* film, which effectively increases the reactive sites and improves the charge transfer efficiency at the interface between the NiO*_x_* film and the electrolyte. The electrochemical results further show that the film electrode (NiO*_x_*-Mg2) has the largest charge storage capacity when the Mg doping concentration is 10%. Compared with the undoped NiO*_x_* film, the doping of Mg improves the transmittance modulation (Δ*T*) performance of the NiO*_x_* film (Δ*T* up to 55.8%) and shortens the response time (2.39 s/0.63 s for coloring/bleaching). In general, Mg doping is an effective method for improving the electrochromic properties of NiO*_x_* films.

## 1. Introduction

Electrochromic materials refer to materials whose optical properties (such as transmittance, absorbance, reflectance, or emissivity) can change dynamically and reversibly under an externally applied low voltage [1,2]. Therefore, they can effectively absorb or reflect external thermal radiation and internal heat diffusion. With their active control ability and low energy consumption characteristics, they are widely used in many fields such as energy-saving smart windows [3,4,5,6], multicolor displays [7,8,9,10], and military camouflage [11,12,13]. Among many inorganic electrochromic materials, NiO*_x_* has been widely studied and applied in electrochromic devices due to its low material cost, high-efficiency performance, and strong anodic coloring optical modulation ability [14,15].

NiO*_x_* film can switch between transparent and dark brown states. Its color-changing mechanism mainly stems from the reversible redox reaction of Ni^2+^ and Ni^3+^ in an alkaline medium [16,17,18]. Currently, the reversible redox reaction of NiO*_x_* is usually represented by Equations (1) and (2) [19]. During the coloring stage, after an external voltage is applied, NiO*_x_* reacts with OH^−^ in the electrolyte, causing the film to turn dark brown. During the bleaching stage, by changing the applied voltage, OH^−^ returns from the film to the electrolyte, and the film then returns to a transparent state. However, pure NiO*_x_* films face many challenges in practical applications, such as long response time, poor light modulation performance, and limited charge storage capacity. These problems severely restrict their wide application in the optoelectronic industry. In view of this, modifying electrochromic materials through various means has become one of the important research directions in the current field.

(1) $$NiO\left(bleached\right)+{OH}^{-}\to NiOOH\left(colored\right)+e^{-}$$

(2) $${Ni\left(OH\right)}_{2}\left(bleached\right)+{OH}^{-}↔NiOOH\left(colored\right)+H_{2}O+e^{-}\;$$

Doping with foreign elements is considered one of the important means to improve the electrochromic performance of NiO*_x_* films [20]. So far, some metal elements have been used to prepare doped NiO*_x_* films. For example, Wang et al. [21] prepared NiO films with different cerium doping concentrations (0.5%, 1%, 2%) using electron beam evaporation technology. Ce doping significantly increased the charge density of the films. The charge density of the 1% Ce: NiO film increased from 4.86 mC·cm^−2^ to 8.14 mC·cm^−2^, an increase of 67.5%. In addition, the 1% Ce: NiO film showed enhanced electrochromic performance. The transmittance modulation (Δ*T*) at 633 nm increased by 27.4%, the coloration efficiency (CE) was enhanced by 27.1%, and the response time was faster (9.64 s/6.76 s for bleaching/coloring). Dang et al. [22] used hydrothermal technology to prepare V-doped NiO films with a two-dimensional porous framework. The introduction of vanadium significantly expanded the surface area of the film, thereby effectively improving the diffusion kinetics of OH^−^ in the electrochromic process. The transmittance modulation was 81.9% at 600 nm, and the response time was improved to 1.2 s/0.9 s (coloring/bleaching time). Zhao et al. [23] introduced Mn into the NiO film to improve its electrochromic performance. The optimized NiO-Mn-3 film (the molar ratio Mn/Ni = 3%) exhibits outstanding electrochromic properties, with particularly significant changes in transmittance modulation (550 nm is 81.8%), fast response time (5.1 s for coloring and 3.5 s for bleaching), and high coloration efficiency (61.09 cm^2^·C^−1^), which are considered to be closely related to the increased specific surface area and enhanced ion diffusion coefficient. These studies clearly reveal the significant influence of metal doping on the electrochromic properties of NiO*_x_* film. Mg, as a lightweight metal, has an ionic radius (Mg^2+^: 0.72 Å) similar to that of (Ni^2+^: 0.69Å) and can introduce defects such as oxygen interstitials or cation vacancies into the NiO*_x_* lattice [24,25,26]. Therefore, the Mg doping strategy is expected to significantly improve the electrochromic properties of NiO*_x_* by optimizing its ion diffusion mechanism.

In this paper, NiO*_x_* films with different Mg doping concentrations (0%, 5%, 10%, 15%) were successfully prepared by the sol–gel method and the spin-coating method, and named NiO*_x_*, NiO*_x_*-Mg1, NiO*_x_*-Mg2, and NiO*_x_*-Mg3, respectively. The lattice strain and defect types induced by Mg doping were systematically analyzed by means of X-ray diffraction, Raman spectroscopy, and X-ray photoelectron spectroscopy. Furthermore, the influence rules of Mg doping on the charge storage capacity, optical modulation ability, and cycling stability of NiO*_x_* films were revealed through cyclic voltammetry, chronoamperometry, and in situ spectroelectrochemical tests. The results show that the NiO*_x_*-Mg2 film electrode exhibits excellent transmittance modulation performance (Δ*T* reaches 55.4%) and a response time of 2.39 s/0.63 s (coloring/bleaching time), which are greatly improved compared with the transmittance modulation (Δ*T* is 46.3%) and response time (coloring/bleaching time was 2.59 s/0.85 s, respectively) of the undoped NiO*_x_* film electrode. In addition, the NiO*_x_*-Mg2 film electrode also shows better charge storage capacity.

## 2. Materials and Experiment Section

### 2.1. Materials

Nickel acetate tetrahydrate (Ni(CH_3_COO)_2_·4H_2_O), ethylene glycol methyl ether (CH_3_OCH_2_CH_2_OH), potassium hydroxide (KOH), and ethanol were purchased from Aladdin Chemical Reagent Co., Ltd. (Shanghai, China). Magnesium acetate tetrahydrate (Mg(CH_3_COO)_2_·4H_2_O), toluene, and acetone were purchased from Sinopharm Chemical Reagent Co., Ltd. (Shanghai, China). The ITO-coated glass was produced by OPV Tech Co., Ltd. (Yingkou, China).

### 2.2. Preparation Method of Precursor

An amount of 0.6221 g of Ni(CH_3_COO)_2_·4H_2_O and 5 mL of ethylene glycol methyl ether were heated at 70 °C with stirring for two hours. Then, the mixture was aged at room temperature for at least 24 h to obtain the NiO_x_ sol precursor. The concentration of the solution was 0.5 mol/L.

The Mg-doped NiO*_x_* sol precursor had a similar preparation method to pure NiO*_x_* sol. Three groups of Mg-doped NiO*_x_* sol were adjusted by changing the addition amount of Mg(CH_3_COO)_2_ 4H_2_O with different doping ratios, maintaining a constant total metal ion concentration of 0.5 mol/L. The doping concentrations of Mg were set at 5%, 10%, and 15%, labeled as NiO*_x_*-Mg1, NiO*_x_*-Mg2, and NiO*_x_*-Mg3, respectively.

### 2.3. Fabrication of Mg-Doped NiO_x_ Films

The ITO substrate (sized 3.0 cm × 1.5 cm) was ultrasonically washed with a foam of deionized water, toluene, acetone, and ethanol. A spin coater was used to coat the substrate surface uniformly with 200 μL NiO*_x_* and Mg-doped NiO*_x_* sol at a speed of 2400 rpm. Then, the substrate was dried on a heating platform at 100 °C for 10 min. Subsequently, the substrate was removed to a muffle furnace for gradient annealing treatment. The annealing process involved increasing the temperature from room temperature to 300 °C at a rate of 5 °C/min, holding it for 60 min, and then allowing it to cool naturally. The above process was repeated three more times. This resulted in NiO*_x_* and Mg-doped NiO*_x_* films.

### 2.4. Characterization

The crystalline phase of the film was characterized by X-ray diffraction (XRD, D8 Advanced, Bruker, Germany) using Cu-k-α radiation. The influence of Mg doping on the crystal structure of NiO_x_ was studied by Raman spectroscopy (DXR2, Thermo Fisher Scientific, America). The microstructure of the NiO*_x_* film doped with magnesium was analyzed by scanning electron microscopy (SEM, Apreo S, Thermo Fisher Scientific, Czech Republic). High-precision spectral data were provided for the surface analysis of Mg-doped NiO*_x_* crystals by X-ray photoelectron spectroscopy (XPS, ESCALAB Xi+, Thermo Fisher Scientific, America). The optical properties of the film were tested using an optical fiber spectrometer (AvaSpec-ULS2048CL-EVO, Avantes, Netherlands). To determine the electrochemical properties of the film, an electrochemical test was carried out using a three-electrode system with the sample as the working electrode, 1M KOH as the electrolyte, a silver/silver chloride (Ag/AgCl) as the reference electrode, and a platinum sheet as the counter electrode. The electrochemical properties of the film were measured using an electrochemical workstation (Squidstat Plus 1454, Admiral, America). The scanning voltage was −0.6 ~ +0.6 V. Cyclic voltammetry (CV) tests were carried out at different scanning rates and with a set number of cycles. The resistance of the film was measured by electrochemical impedance spectroscopy (EIS), and the electrochemical properties of the film were analyzed.

## 3. Results and Discussion

### 3.1. Sample Characterization

**Figure 1:**
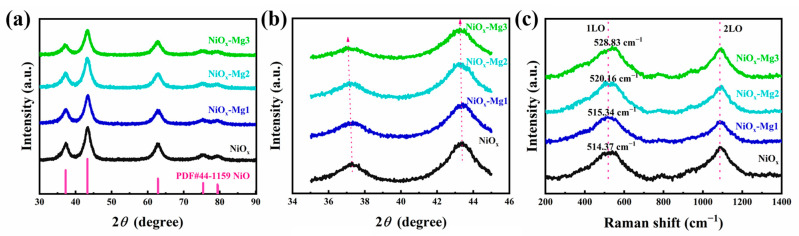
(**a**) XRD patterns of NiO*_x_*, NiO*_x_*-Mg1, NiO*_x_*-Mg2, and NiO*_x_*-Mg3 films. (**b**) XRD local amplification diagram. (**c**) Raman spectra of the Mg-doped NiO*_x_* films.

The effect of Mg doping on the crystal structure of NiO*_x_* films was systematically investigated using X-ray diffraction (XRD) technology. As shown in Figure 1a, the XRD patterns of all samples exhibited similar diffraction peak characteristics, and obvious characteristic peaks were detected at 2θ = 37.2°, 43.2°, 62.9°, 75.4°, and 79.3°, corresponding to the (101), (012), (110), (113), and (202) crystal planes in the PDF#44-1159 card, respectively. Through the analysis of the XRD patterns, it was determined that all samples presented a typical NiO*_x_* face-centered cubic structure, and no impurity phase was detected, indicating that Mg doping did not introduce any impurity phases. In addition, compared with undoped NiO*_x_* films, the diffraction peaks of Mg-doped films shifted slightly toward small angles (see Figure 1b), indicating that the interplanar spacing of the crystal planes of Mg-doped films increased slightly. This can be attributed to the difference in ionic radius between Ni^2+^ (0.69 Å) and Mg^2+^ (0.72 Å) [23]. Furthermore, the Raman spectra of NiO*_x_* and its Mg-doped NiO*_x_* films were characterized, and the results are shown in Figure 1c. The Raman spectra of the four films exhibit two characteristic peaks located at 514.37 cm^−1^ and 1090 cm^−1^, respectively. These two characteristic peaks correspond to the single-phonon longitudinal optical (1LO) mode and the two-phonon longitudinal optical (2LO) mode [27]. In the Raman spectrum of the undoped NiO*_x_* film, the broad peak at 514.37 cm^−1^ is consistent with the reports in the literature regarding the NiO*_x_* phase. This peak is due to the Ni-O stretching vibration mode and the single-phonon longitudinal optical mode in NiO*_x_* [28]. It is worth noting that the appearance of the broad 1LO peak in the film’s Raman spectrum may be related to nickel vacancies or oxygen interstitial defects, which may disrupt the periodicity of the lattice, causing the LO phonon mode to no longer strictly satisfy momentum conservation, resulting in peak displacement and broadening [29,30]. As the Mg doping concentration gradually increases, the 1LO peak shows an obvious blue shift. When the Mg doping concentrations are 5%, 10%, and 15%, respectively, the 1LO peak blue-shifts to 515.34 cm^−1^, 520.16 cm^−1^, and 528.83 cm^−1^, respectively. In addition, we systematically characterized the intrinsic optical transmittance spectra of NiO*_x_* and Mg-doped NiO*_x_* films using ordinary slides as the substrate, and accurately calculated their optical band gaps (*E*_g_) based on the Tauc plot. It was found that the *E*_g_ of films showed a monotonically increasing trend with the doping concentration gradient. As shown in Figure S1, the optical band gap of the undoped sample is 3.25 eV. As the Mg doping amount gradually increases (NiO*_x_*-Mg1, NiO*_x_*-Mg2, and NiO*_x_*-Mg3), its *E*_g_ values correspond to 3.27 eV, 3.30 eV, and 3.34 eV, respectively, showing a significant doping concentration dependence. This phenomenon is attributed to the lattice expansion induced by Mg doping: the substitution of Ni^2+^ (0.69 Å) by Mg^2+^ (0.72 Å) causes local lattice distortion, leading to an upward shift in the conduction band minimum (CBM) energy level. At the same time, the formation of oxygen interstitials further expands the energy separation between the valence band maximum (VBM) and the conduction band by enhancing the electron–phonon coupling effect [31], thereby reducing the absorption of the NiO*_x_* film in the visible light region and improving its optical transparency in the bleached state. 

**Figure 2:**
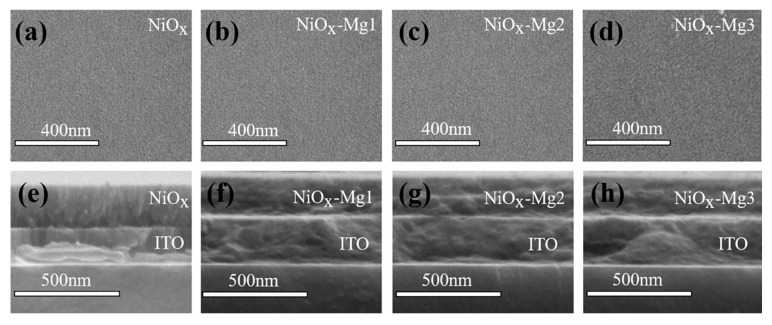
Surface (**a**–**d**) and cross-sectional SEM images (**e**–**h**) of NiO*_x_* and Mg-doped NiO*_x_* films.

Figure 2a–h show scanning electron microscope (SEM) images of the surface and cross-sectional morphology of undoped and Mg-doped NiO*_x_* films. From the surface SEM images in Figure 2a–d, it can be seen that all films exhibit a uniform and flat surface morphology. Further, the cross-sectional morphology of the films was characterized, and the results are shown in Figure 2e,f. The thicknesses of NiO*_x_*-Mg1, NiO*_x_*-Mg2, and NiO*_x_*-Mg3 are 242 nm, 248 nm, and 240 nm, respectively, while the thickness of the undoped NiO*_x_* film is 258 nm. In addition, the undoped NiO*_x_* film presents a relatively dense microstructure. In contrast, the microstructures of the NiO*_x_*-Mg1, NiO*_x_*-Mg2, and NiO*_x_*-Mg3 films are relatively looser. This relatively loose morphological feature is beneficial to the ion intercalation and deintercalation processes, thus helping to improve the electrochromic performance of the films. The energy-dispersive spectroscopy (EDS) elemental distribution map shows that the Ni, O, and Mg elements are uniformly distributed in NiO*_x_*-Mg1, NiO*_x_*-Mg2, and NiO*_x_*-Mg3 (Figure S2a–d), and the quantitative analysis results of the elemental composition are summarized in Table 1. According to the EDS data, the atomic percentages of Mg elements in NiO*_x_*-Mg1, NiO*_x_*-Mg2, and NiO*_x_*-Mg3 increase successively, being 1.9%, 3.0%, and 4.6%, respectively. This trend fully confirms the effectiveness of the Mg doping concentration gradient design. Further analysis finds that the Mg/(Mg + Ni) ratios of NiO*_x_*-Mg1, NiO*_x_*-Mg2, and NiO*_x_*-Mg3 are 5.4%, 9.6%, and 14.6%, respectively, which are close to the theoretical doping concentrations (5%, 10%, and 15%, respectively) [32]. Compared with undoped NiO*_x_*, the nickel content in NiO*_x_*-Mg2 and NiO*_x_*-Mg3 significantly decreased to 28.1% and 27.0%, while the oxygen content slightly decreased to 68.9% and 68.4%. The decrease in oxygen element is much lower than the loss of nickel, indicating the existence of oxygen interstitial defects during the Mg doping process. In addition, the decrease in oxygen element content also indicates the generation of oxygen vacancy defects. Relevant studies have shown that the introduction of an appropriate amount of oxygen defects helps to enhance the electrochromic performance of the film [33,34].

**Figure 3:**
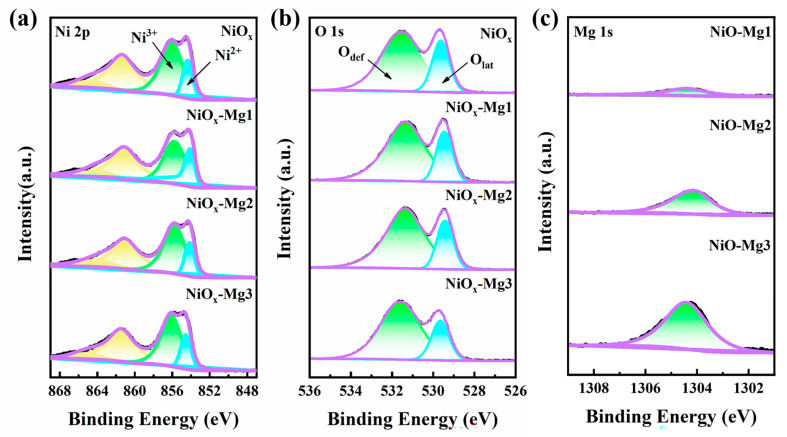
XPS spectra of NiO*_x_* and Mg-doped NiO*_x_* films: (**a**) Ni 2p, (**b**) O 1s, and (**c**) Mg 1s.

To explore the surface composition and chemical state of the films, XPS measurements were carried out on the original NiO*_x_* and Mg-doped NiO*_x_* films. As shown in Figure 3, the characteristic satellite peak of Ni 2p appears near 862 eV, confirming that nickel exists in the form of a mixed valence state (Ni^2+^/Ni^3+^), which is consistent with the electronic structure characteristics of typical nickel oxides [19]. The results show that the binding energies of Ni^2+^ and Ni^3+^ of the intrinsic NiO*_x_* are located at 854.35 eV and 856.05 eV, respectively (see Table S1). With the change in the Mg doping concentration gradient, the Ni 2p peak of the low-doping groups (NiO*_x_*-Mg1 and NiO*_x_*-Mg2) shows a negative shift of 0.2–0.4 eV, while the high-doping group (NiO*_x_*-Mg3) shows a positive shift of about 0.3 eV. In addition, as the doping amount of Mg^2+^ increases, the ratio of Ni^3+^/Ni^2+^ shows an upward trend. This might be due to the fact that Mg ions caused some nickel vacancies during the doping process. To maintain charge neutrality, some Ni^2+^ was oxidized to Ni^3+^, thereby increasing the relative content of Ni^3+^ [19,35]. O 1s can be decomposed into two main components: lattice oxygen (O_lat_) and oxygen defects (O_def_) [36]. The peak located at 529.55 eV is attributed to O_lat_, indicating that O forms a stable oxide structure with Ni; the peak located at 531.50 eV is attributed to O_def_ (oxygen interstitials and oxygen vacancies), corresponding to the O^2−^ state in the oxygen defect region [37]. By calculating the ratio of O_def_/O_lat_ (Table S1), it is found that the ratio of O_def_/O_lat_ gradually increases with the increase in the doping ratio, indicating that Mg doping introduced oxygen interstitials and oxygen vacancies in the NiO_x_ film. The Mg 1s peak is located at 1304.5 eV, indicating that Mg ions are successfully doped into the nickel oxide film.

### 3.2. Electrochemical and Electrochromic Properties

**Figure 4:**
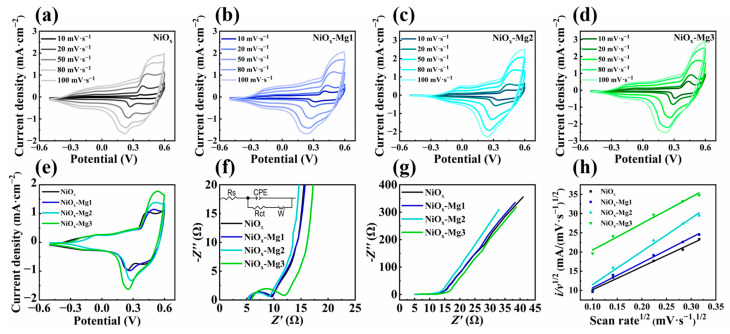
Cyclic voltammetry (CV) curves of (**a**–**d**) NiO*_x_* and Mg-doped NiO*_x_* films at different scan rates. (**e**) The cyclic voltammetry curves of NiO*_x_* and Mg-doped NiO*_x_* films at 50 mV·s^−1^ scan rate were compared. Electrochemical impedance spectroscopy of NiO*_x_* and Mg-doped NiO*_x_* thin films in the high-frequency region (**f**) and low-frequency region (**g**). The illustration in (**f**) is an equivalent circuit diagram. (**h**) The *i*/*v*^1/2^-*v*^1/2^ curves of NiO*_x_* and Mg-doped NiO*_x_* films were obtained.

The electrochemical behaviors of the original NiO*_x_* and Mg-doped NiO*_x_* electrode systems were systematically characterized by cyclic voltammetry (CV). The experiment was carried out in the 1 M KOH electrolyte system, with the potential window set from −0.6 V to 0.6 V (vs. reference electrode) and the scanning rate gradient set at 10–100 mV·s^−1^. As shown in Figure 4a–d, with the increase in the scanning rate, the integral area of the CV curve showed an upward trend, and the anodic oxidation peak potential shifted positively. This is because at a high scanning rate, the rapid polarization of the electric double layer at the electrode–electrolyte interface causes the mass transfer rate to lag behind the electron transfer rate [38]. Further comparative analysis of CV at the 50 mV·s^−1^ scanning rate (Figure 4e) revealed that with the increase in the Mg doping concentration, the integral area of the CV curve of the electrode material showed a significant increasing trend, accompanied by the displacement of the redox characteristic peaks. This is because Mg doping induces the formation of oxygen defects, significantly increasing the density of active sites on the surface and leading to an increase in the integral charge.

The regulation mechanisms of the interface transport characteristics and the ion diffusion behavior of NiO*_x_* electrode materials doped with different Mg concentrations were thoroughly investigated by electrochemical impedance spectroscopy. Figure 4f shows the built-in equivalent circuit, where *R*_s_ represents the ohmic resistance of the electrolyte and *R*_ct_ corresponds to the charge transfer resistance in the semicircular region; the smaller the radius, the lower the transfer resistance. The linear portion in the low-frequency region is described by the Warburg element *W*, and an increase in slope indicates faster ion diffusion. The parallel CPE element reflects the double-layer capacitance behavior at the interface [39]. The quantitative analysis of the Nyquist plots in the high-frequency region indicates that all samples exhibit similar capacitive arc characteristics. Among them, the NiO*_x_*-Mg2 sample has the smallest semicircle radius, which indicates the lowest charge transfer resistance. However, the NiO*_x_*-Mg3 sample may cause lattice distortion due to excessive doping, resulting in a substantial increase in the interface charge transfer resistance. Further analysis of the Warburg impedance behavior in the low-frequency region of Figure 4g reveals that compared with the NiO*_x_* electrode, the slope of the NiO*_x_*-Mg2 electrode shows an increasing trend, while that of the NiO*_x_*-Mg3 electrode shows a decreasing trend. The results show that the NiO*_x_*-Mg2 electrode has relatively optimal ion transport efficiency. Moderate Mg doping increases the density of active sites on the electrode surface, thereby enhancing the charge storage and release response rate of the NiO*_x_* electrode. In contrast, excessive doping may increase the ion diffusion barrier due to lattice distortion, thereby inhibiting ion transport.

**Figure 5:**
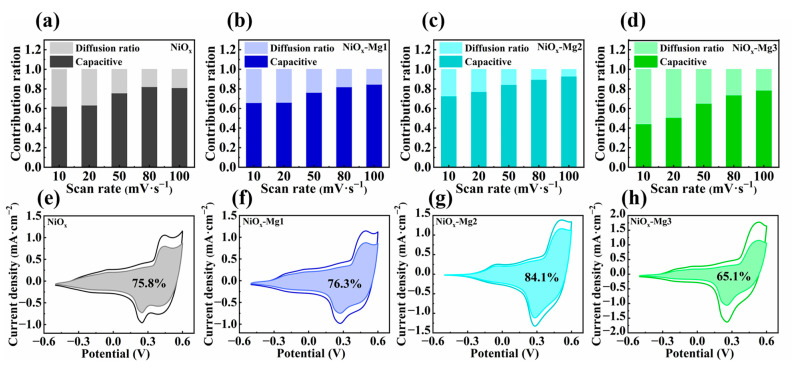
The capacitance contribution and diffusion contribution of (**a**–**d**) NiO*_x_* and Mg-doped NiO*_x_* films at different scan rates. The pseudocapacitance ratio of (**e**–**h**) NiO*_x_* and Mg-doped NiO*_x_* films at 50 mV·s^−1^ scan rate.

Based on Equation (3), we use CV data to quantitatively analyze the contribution of pseudocapacitance and simulate the reaction kinetics accordingly [40]:

(3) $$i_{v}=k_{1}v+k_{2}v^{1/2}$$

where *i_v_* is the anodic peak current, *v* is the scanning rate, and *k*_1_ and *k*_2_ are constants. Figure 4h shows the *i*/*v*^1/2^-*v*^1/2^ curves of different electrode materials. By calculating *k*_1_ and *k*_2_, we can distinguish the contribution rates of the capacitive effect (*k*_1_*v*) and diffusion controlled insertion (*k*_2_*v*^1/2^) at a specific potential. Figure 5a–d show the quantitative analysis results of the capacitance contribution ratio of electrode materials with different doping ratios under the gradient change in voltametric scanning rate. The experimental data show that all samples follow the scanning rate dependence rule: as the scanning rate (10–100 mV⋅s^−1^) increases, the proportion of rapid redox reactions driven by electrochemical polarization on the electrode surface increases significantly, resulting in a systematic upward trend in the proportion of the interfacial pseudocapacitance effect. It is worth noting that the low-doping systems (NiO*_x_*-Mg1, NiO*_x_*-Mg2) show a higher proportion of pseudocapacitance contribution than the original NiO*_x_* electrode in the entire scanning rate range, while the capacitance contribution value of the high-doping sample (NiO*_x_*-Mg3) is lower than that of the undoped system. Further, through the quantitative decomposition at the 50 mV·s^−1^ scanning rate (Figure 5e–h), it is found that the proportions of pseudocapacitance contribution of the NiO*_x_*, NiO*_x_*-Mg1, NiO*_x_*-Mg2, and NiO*_x_*-Mg3 electrodes are 75.8%, 76.3%, 84.1%, and 65.1%, respectively. This data pattern reveals a strong correlation between the doping concentration and the charge storage mechanism: moderate Mg doping (NiO*_x_*-Mg1, NiO*_x_*-Mg2) significantly enhances the surface-controlled pseudocapacitance behavior by optimizing the density of active sites on the electrode surface, which can enhance the charge storage and release response rate, while excessive doping (NiO*_x_*-Mg3) may increase the ion diffusion barrier due to lattice distortion, resulting in a gradual transformation of the charge storage process to the bulk diffusion-controlled mechanism [41].

**Figure 6:**
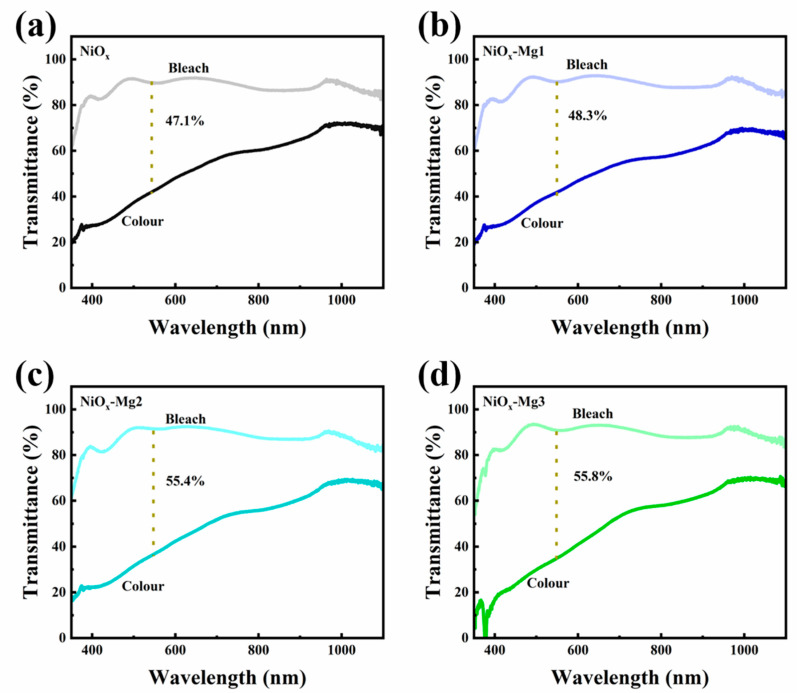
In situ transmission spectra of (**a**) NiO*_x_* and (**b**–**d**) Mg-doped NiO*_x_* films at +0.6 V/−0.6 V potential.

The transmittance modulation properties of the electrode materials in the colored and bleached states were systematically characterized by ultraviolet–visible–near-infrared spectrophotometry. As shown in Figure 6a–d, the Δ*T* of the original NiO*_x_* electrode is 47.1% at 500 nm (characteristic wavelength). After Mg doping modification, the transmittance modulation properties of NiO*_x_*-Mg1, NiO*_x_*-Mg2, and NiO*_x_*-Mg3 electrodes were significantly improved, and their Δ*T* values reached 48.3%, 55.4%, and 55.8%, respectively. This improvement in performance is mainly due to the regulatory effect of Mg doping on the energy band structure of the material: the defect states introduced by doping increase the charge injection amount and improve the redox activity during the electrochromic process, thereby improving the light absorption efficiency. It is worth noting that as the doping concentration continues to rise, the transmittance modulation performance shows a trend of rapid improvement first and then saturation, indicating the existence of an optimal doping concentration threshold.

**Figure 7:**
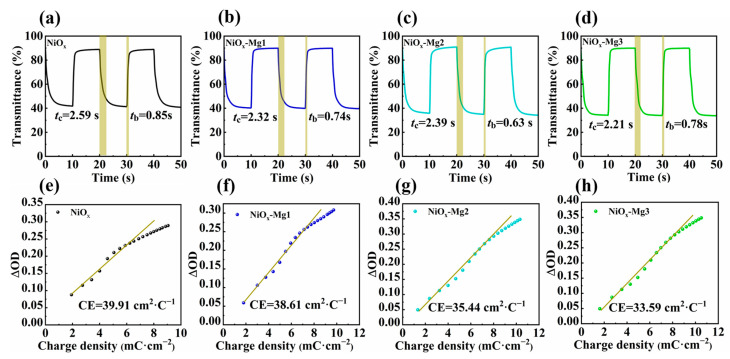
The transmittance–time response curves of (**a**–**d**) NiO*_x_* and Mg-doped NiO*_x_* films at 500 nm are shown. Coloring efficiency of (**e**–**h**) NiO*_x_* and Mg-doped NiO*_x_* films.

Using the chronoamperometry method combined with ultraviolet–visible–near-infrared spectroscopy technology, the dynamic response characteristics of the electrode materials were systematically studied. As shown in Figure 7a–d, the coloring response time (*t*_c_) and bleaching response time (*t*_b_) of the original NiO*_x_* electrode were 2.59 s and 0.85 s, respectively. After doping modification with Mg, the response times (*t*_c_/*t*_b_) of the NiO*_x_*-Mg1, NiO*_x_*-Mg2, and NiO*_x_*-Mg3 electrodes were optimized to 2.32 s/0.74 s, 2.39 s/0.63 s, and 2.21 s/0.78 s, respectively, indicating that Mg doping effectively shortened the response time of charge storage and release of the NiO*_x_* electrode by optimizing the density of active sites on the electrode surface, thereby significantly improving its response rate. Another key indicator for measuring the electrochromic performance of nickel oxide films is the coloration efficiency (CE). Its physical meaning is the change in optical density (ΔOD) caused by the insertion/extraction of charge per unit area, which can also be understood as the ratio of the optical contrast between the colored and bleached states of the film at a fixed wavelength to the charge density [42]. The formula is as follows:

(4) $$CE=\frac{∆OD}{∆Q}=\frac{\log{(T}_{b}/T_{c})}{Q/A}$$

where *Q* refers to the amount of charge embedded or extracted per unit area and *A* is the electrode area. *T*_b_ and *T*_c_ are the transmittances of the film in the bleached state and the colored state, respectively. Based on the test results of response time and optical modulation, the influence law of Mg doping on the CE of NiO*_x_* electrode materials was systematically studied, as shown in Figure 7e–h. The experimental results show that the CE value of the original NiO*_x_* electrode is 39.91 cm^2^⋅C^−1^, while the CE values of the doped samples NiO*_x_*-Mg1, NiO*_x_*-Mg2, and NiO*_x_*-Mg3 are reduced to 38.61 cm^2^⋅C^−1^, 35.44 cm^2^⋅C^−1^, and 33.59 cm^2^⋅C^−1^, respectively, showing an obvious downward trend dependent on the doping concentration. This phenomenon is in sharp contrast to the improvement in the optical modulation performance, revealing the multiple influence mechanisms of doping on the electrochromic performance. The decrease in the CE value may be attributed to the following factors: although the defects introduced by doping enhance the light absorption, they may also increase the carrier scattering and reduce the effective charge utilization rate; doping may alter the energy band structure of the material and affect the reversibility of the redox reaction [43,44]. It is worth emphasizing that although the CE value decreases with the increase in doping, its decrease amplitude (16%) is less than the improvement amplitude of the optical modulation performance (18.5%), indicating that doping still has a positive effect on the overall performance optimization.

**Figure 8:**
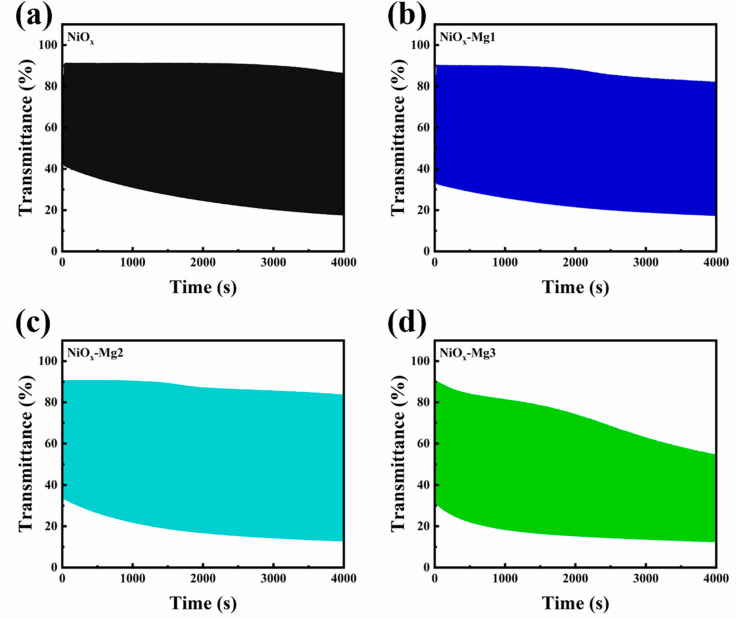
The transmittance curves of (**a**) NiO*_x_* and (**b**–**d**) Mg-doped NiO*_x_* films with the number of cycles.

A systematic study based on the potentiostatic polarization method was performed to comprehensively evaluate the influence of Mg doping on the cycling stability of NiO*_x_* electrode materials. The double-potential step method was used in the experiment (coloring state: +0.6 V vs. Ag/AgCl; bleaching state: −0.6 V vs. Ag/AgCl), and each potential was maintained for 10 s to guarantee that the system reached a steady state. As shown in Figure 8, the pristine NiO*_x_* electrode exhibited excellent cycling stability within the test range of 4000 s. Slight performance degradation of the lightly doped samples (NiO*_x_*-Mg1, NiO*_x_*-Mg2) began to occur around 2000 s, while the heavily doped sample (NiO*_x_*-Mg3) showed significant performance degradation from the beginning, and the degradation rate increased with time. The light absorption of all samples in the colored state showed a gradually increasing trend, which might be attributed to the gradual activation of active sites on the electrode surface during the cycling process [45].

## 4. Conclusions

In this work, we successfully prepared NiO*_x_* films with different Mg doping concentrations by the sol–gel method and the spin-coating method, and systematically studied their structure, electrochemical and electrochromic properties. The results show that the surface of the Mg-doped film is uniform and smooth, and the microstructure tends to be loose, which is beneficial to the insertion and extraction of OH^−^. At the same time, we found that Mg doping can introduce oxygen defects in the NiO*_x_* lattice, effectively increase the reactive sites of the film electrode, and improve the charge transfer efficiency at the interface between the film electrode and the electrolyte. In addition, the charge storage capacity and electrochromic properties Mg-doped NiO*_x_* films can be effectively improved. Among them, the NiO*_x_*-Mg2 film electrode exhibits excellent transmittance modulation (Δ*T* reaches 55.4%) and response time (2.39 s/0.63 s for coloring/bleaching). In general, Mg doping effectively improves the electrochromic properties of NiO*_x_* films by optimizing their ion diffusion mechanism. This will provide a reference for the development of efficient energy storage electrochromic devices.

## Figures and Tables

**Table 1:** The element proportions of Mg-doped NiO*_x_* films.

Sample	Ni (%)	O (%)	Mg (%)	Mg/(Mg + Ni) (%)
NiOx	30.3	69.7	0	0
NiOx-Mg1	32.9	65.2	1.9	5.4
NiOx-Mg2	28.1	68.9	3.0	9.6
NiOx-Mg3	27.0	68.4	4.6	14.6

## Data Availability

The data that support the findings of this study are available from the corresponding author upon reasonable request.

## Supplementary Materials

The following supporting information can be downloaded at: https://www.mdpi.com/article/10.3390/nano15161217/s1, Figure S1: The in-situ transmittance curves (a) and Tauc plots (b) of NiO*_x_* and Mg-doped NiO*_x_* films; Figure S2: The EDS diagram of NiO*_x_* (a), NiO*_x_*-Mg1 (b), NiO*_x_*-Mg2 (c) and NiO*_x_*-Mg3 (d) films; Figure S3: Current-time response of (a-d) NiO*_x_* and Mg-doped NiO*_x_* films. Table S1: The positions of Ni 2p and O 1s and the proportion of oxygen defects in NiO*_x_* and Mg-doped NiO*_x_* films were investigated.
