# Supplementary material for: Enhanced Electrochromic Properties of NiOx Films Through Magnesium Doping Strategy
Source: Nanomaterials (Basel). 2025 Aug 8;15(16):1217. doi: 10.3390/nano15161217 (PMC12388670; doi:10.3390/nano15161217)
Supplement: Supplementary file 1 [file nanomaterials-15-01217-s001.zip › nanomaterials-3784894-supplementary.pdf]

Enhanced electrochromic properties of NiO_x films through Magnesium doping strategy

Xiaoyu Yao [#,1], Shuai Ding [#,1], Xiaoyu Shen [1], Congkai Guo [1], Yao Liu [1], Wenjuan Xia [1], Guohua Wu [,2,3] and Yaohong Zhang [*,1]*

[1] School of Physics, Northwest University, Xi'an 710127, China

[2] Qingdao Innovation and Development Center of Harbin Engineering University, Harbin Engineering University, Qingdao 266000, China.

[3] College of Materials Science and Chemical Engineering, Harbin Engineering University, Harbin 150001, China.

∗ Correspondence: ghwu@hrbeu.edu.cn (G.W.); yhzhang@nwu.edu.cn (Y.Z.)

[#] These authors contributed equally to this work.

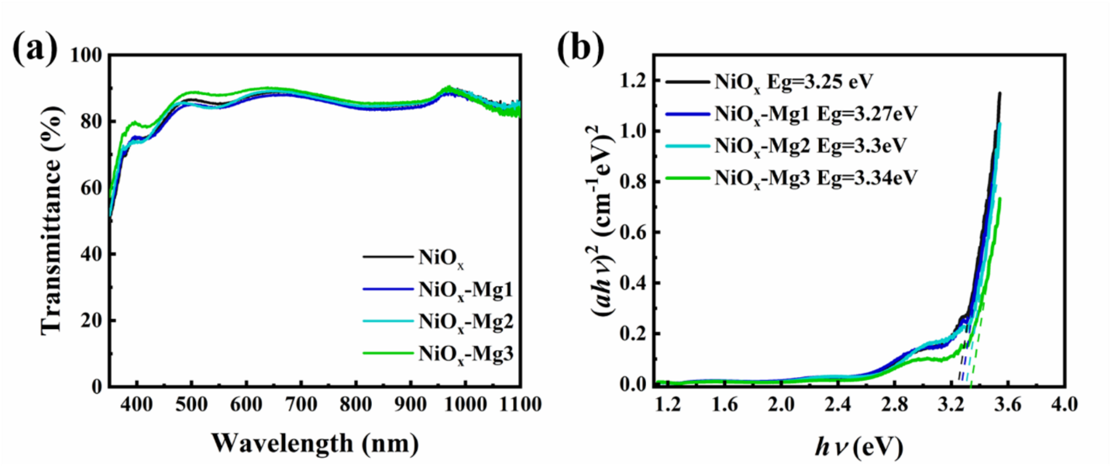

Figure S1. The in-situ transmittance curves (a) and Tauc plots (b) of NiO_x and Mg-doped NiO_x films.

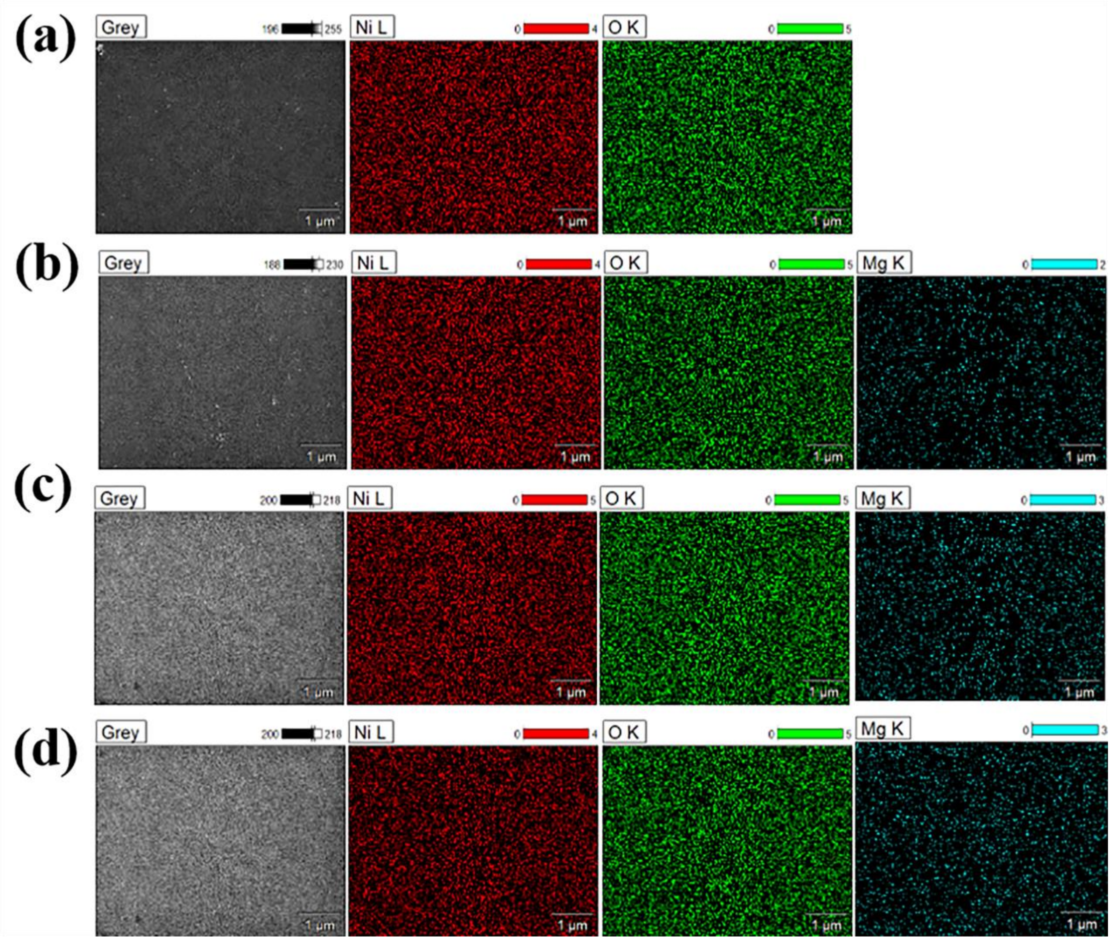

Figure S2. The EDS diagram of NiO_x (a), NiO_x-Mg1 (b), NiO_x-Mg2 (c) and NiO_x-Mg3 (d) films.

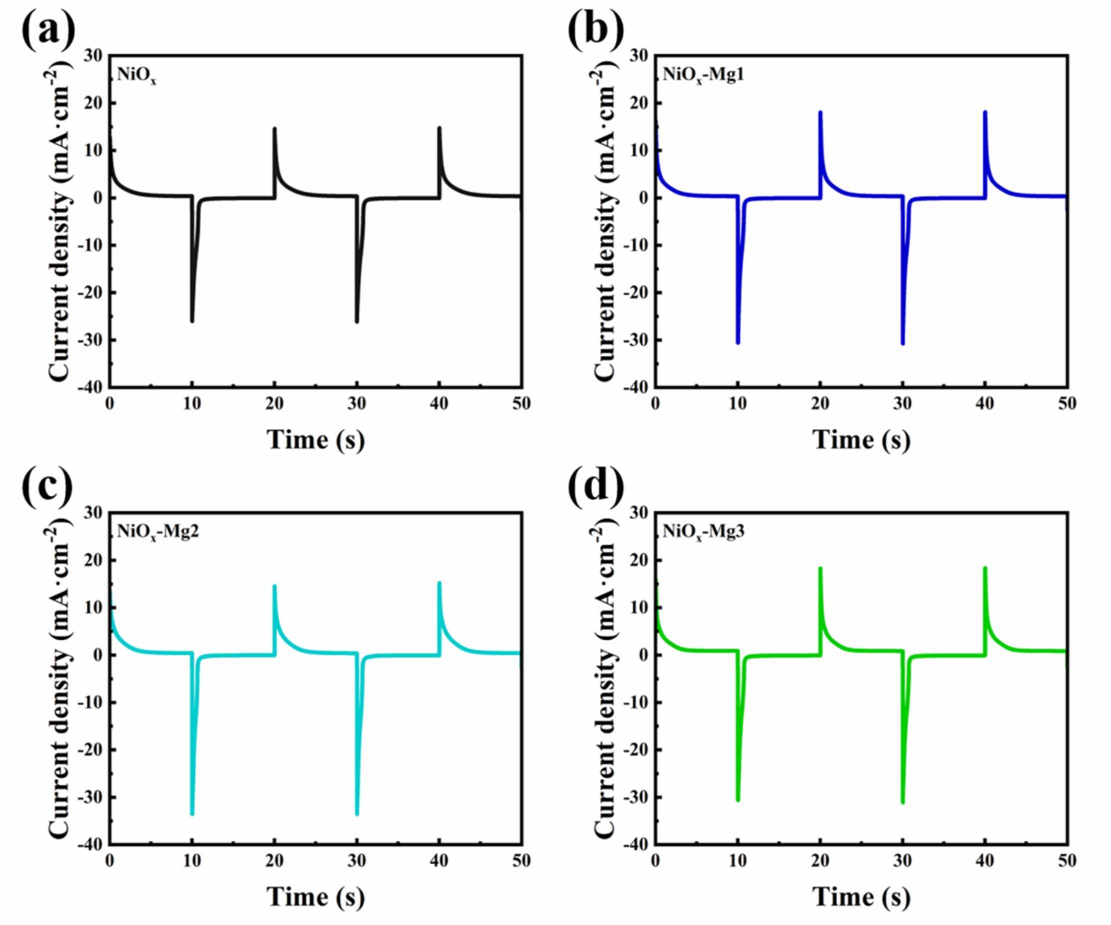

Figure S3. Current-time response of (a) NiO_x and (b-d) Mg-doped NiO_x films.

Table S1. The positions of Ni 2p and O 1s and the proportion of oxygen defects in NiO_x and Mg-doped NiO_x films were investigated.

Sample	NiO_x	NiO_x-Mg1	NiO_x-Mg2	NiO_x-Mg3
Ni^{2+} (eV)	854.35	854.1	854.15	854.55
Ni^{3+} (eV)	856.05	855.8	855.75	856.15
O_{lat} (eV)	529.55	529.4	529.42	529.65
O_{def} (eV)	531.5	531.35	531.35	531.6
Area ratio (O_{def}/O_{lat})	269.7%	289.1%	294.6%	308.2%
Area ratio (Ni^{3+}/ Ni^{2+})	222.2%	230.6%	280.8%	305.6%
